# Complete Genome Sequences of Two Cutibacterium acnes Strains Isolated from an Orthopedic Surgical Site

**DOI:** 10.1128/MRA.00290-20

**Published:** 2020-04-23

**Authors:** Kaito Seo, Kazuki Tanaka, Shinji Fukuda, Kazuharu Arakawa

**Affiliations:** a Institute for Advanced Biosciences, Keio University, Tsuruoka, Japan; b Systems Biology Program, Graduate School of Media and Governance, Keio University, Fujisawa, Japan; c Intestinal Microbiota Project, Kanagawa Institute of Industrial Science and Technology, Kawasaki, Japan; d Transborder Medical Research Center, University of Tsukuba, Tsukuba, Japan; e Faculty of Environment and Information Studies, Keio University, Fujisawa, Japan; Portland State University

## Abstract

Cutibacterium acnes was reported as one of the most frequently isolated opportunistic pathogens among the skin microbiota. Here, we report two complete genome sequences of Cutibacterium acnes strains that were isolated from surgical (strain SZ1) and nonsurgical (strain SZ2) sites on the back of an orthopedic surgery patient.

## ANNOUNCEMENT

Cutibacterium acnes is a lipophilic anaerobic Gram-positive commensal skin bacterium, often implicated in surgical site infections. It is inferred that contamination derives mainly from the skin adjacent to the surgical wound (1). While there is no specific method of prevention to avoid the infections caused by *C. acnes* (1), genomic information may provide clues for establishing effective preventative measures.

*C. acnes* was isolated as a single colony from surgical (strain SZ1) and nonsurgical (strain SZ2) sites on an orthopedic patient onto a Gifu anaerobic medium (GAM) agar medium plate and cultured under anaerobic conditions. Species identification was confirmed based on the 16S rRNA gene, amplified with *C. acnes* 16S rRNA gene-specific PCR, run using the primers CA_F and CA_R, which target positions 414 to 1445, resulting in a BLAST identity of 100% to a previously sequenced *C. acnes* strain. The colonies were incubated with GAM liquid medium and cultured for 2 days at 37°C under anaerobic conditions prior to the isolation of genomic DNA using a Genomic-tip 20/G (Qiagen), following the manufacturer’s protocol. Both the Nanopore and Illumina sequencing libraries were prepared from the same genomic DNA. Long-read sequencing libraries were prepared using the rapid barcoding sequencing protocol (SQK-RBK004) and sequenced on a GridION device with a FLO-MIN 106 flow cell (Oxford Nanopore Technologies), yielding 155,000 (*N*_50_ length, 10 kbp) and 131,000 (*N*_50_ length, 6 kbp) reads for strains SZ1 and SZ2, respectively. Illumina sequencing was performed for error correction by using a HyperPlus kit (Kapa Biosystems) for library preparation and a NextSeq 500 sequencer using high-output mode and 75 cycles (Illumina), yielding 49 million and 85 million reads for strains SZ1 and SZ2, respectively. Unfiltered Nanopore reads with at least 5,000 bp and all reads were used for the *de novo* assembly of strains SZ1 and SZ2, corresponding to 125-fold and 74-fold coverages, respectively. Using Canu v1.8.0 (2) with default parameters, the genomes were assembled into single contigs and were manually circularized by deleting the overlapping ends through a BLAST search of the first 10 kbp within the genome, prior to Pilon polishing. The assembled chromosomes were further polished by mapping the raw Illumina short reads using the Burrows-Wheeler Aligner v0.7.17 with MEM algorithm (3), sorted and indexed with SAMtools (4), and finally error corrected using Pilon (5). The assembly completeness was assessed using BUSCO v1 (6) with the gVolante server (7), and the genomes were annotated using the DFAST server (8), as previously described (9). The two draft genome sequences of *C. acnes* have 100% BUSCO completeness and span 2,494,525 bp (SZ1) and 2,504,552 bp (SZ2), with a GC content of 60.0%. The genomes were predicted to contain 2,225 to 2,236 putative coding sequences, respectively. Default parameters were used for all software unless otherwise noted.

For analysis of the lineage type of the two *C. acnes* strains, nine housekeeping genes (*aroE*, *atpD*, *camp2*, *gmk*, *guaA*, *lepA*, *recA*, *sodA*, and *tly*) were checked on the Cutibacterium acnes multilocus sequence typing (MLST) website (https://pubmlst.org/cacnes/) (10). Both isolates had the allelic profile of 1-1-1-1-3-1-1-1-1 and were classified as type IA_1_ based on the MLST scheme (11). McDowell et al. reported that the type IA_1_ group consisted of 85% antibiotic-resistant isolates in their analysis (11). Therefore, the strain obtained from the surgical site could have survived the sterilization protocol using antibacterial substances.

This study was approved by the ethics committee of Keio University School of Medicine under approval number 20150102. The subject was informed of the purpose of this study, and written consent was obtained from the subject.

### Data availability.

The complete genome sequences have been deposited in DDBJ under the accession numbers AP022844 and AP022845 and in the Sequence Read Archive (SRA) under BioProject accession numbers PRJNA608175 and PRJNA608176 for strains SZ1 and SZ2, respectively.
